# Association between cardiometabolic index and albuminuria: Evidence from NHANES 2017–2020

**DOI:** 10.1371/journal.pone.0318736

**Published:** 2025-02-25

**Authors:** Qiming Xu, Junyan Lin, Lin Liao, Jing Hu, Jianrao Lu

**Affiliations:** 1 Institute of Kidney Disease of Shanghai University of Traditional Chinese Medicine, Shanghai, China; 2 Department of Nephrology, Seventh People’s Hospital Affiliated to Shanghai University of Traditional Chinese Medicine, Shanghai, China; Memorial Sloan Kettering Cancer Center, UNITED STATES OF AMERICA

## Abstract

**Introduction:**

Albuminuria is a crucial marker of kidney damage and serves as an early indicator of the risk for chronic kidney disease (CKD). Recent studies have suggested that the cardiometabolic index (CMI), could be valuable for screening renal insufficiency. However, the relationship between CMI and albuminuria remains underexplored. Therefore, the aim of this study was to investigate the association between CMI and albuminuria, with the goal of providing new insights for the clinical diagnosis, assessment, and early intervention of kidney disease.

**Methods:**

The National Health and Nutrition Examination Survey (NHANES) for the period between 2017–2020 provided the data for this cross-sectional investigation. Triglyceride (TG) (mmol/L)/High density lipid-cholesterol (HDL-C) (mmol/L) ×  Waist height ratio (WHtR) was the formula used for calculating CMI. Using multifactorial logistic regression, the independent connection between albuminuria and CMI was investigated. The threshold effect was determined by means of a two-stage linear regression model. Additionally, subgroup analysis and interaction tests were carried out.

**Results:**

A total of 3,339 participants were included, and 12.38% of them had albuminuria. As the CMI quartiles grew (quartile 1: 7.78%, quartile 2: 13.43%, quartile 3: 12.93%, quartile 4: 17.01%), so did the probability of albuminuria. The results of adjusted model 3 showed that a greater probability of albuminuria prevalence was strongly correlated with CMI (OR =  2.26, 95% CI: 1.58–3.23). This association held true for all subgroups (all P for trend >  0.05). Furthermore, with a two-stage linear regression model with an inflection point of 0.92, we discovered a nonlinear relationship between CMI and albuminuria.

**Conclusions:**

Our findings indicate that CMI levels are significantly associated with the risk of albuminuria prevalence, suggesting that CMI could serve as a valuable biomarker for assessing the risk of albuminuria.

## 1 Introduction

Chronic kidney disease (CKD) is defined as the presence of abnormalities in kidney structure or function that persist for at least three months and significantly impact health [1]. It is typically characterized by a decline in estimated glomerular filtration rate (eGFR) or an elevation in urinary albumin levels [2]. The prevalence of CKD is as high as 9.1% worldwide, according to JAMA intern Med 2023. Approximately 10% of CKD patients will eventually develop end stage renal disease (ESRD) [3], while more than 2 million patients will pass away each year as a result of not receiving treatment [4]. It is now a global public health issue that poses a major threat to human health. Therefore, the identification of specific biomarkers for the early diagnosis of CKD is crucial, as it would facilitate earlier intervention and contribute to more effective prevention strategies [5].

Since increased albuminuria is the most sensitive and accurate diagnostic indication for early renal disease, it has been widely employed [6]. Patients with CKD are commonly associated with albuminuria, and the presence of urinary proteins is closely linked to lipid metabolism disturbances. Epidemiological and pathophysiological studies have indicated that elevated lipid levels in urine may serve as an early marker of CKD. Additionally, urinary albumin further exacerbates renal injury and disrupts lipid metabolism. Dysregulated lipid metabolism induces chronic low-grade systemic inflammation and oxidative stress, which contribute to the progression of proteinuria [7–10]. However, accurately quantifying these processes remains challenging when using traditional markers of lipid metabolism [11]. Ichiro Wakabayashi et al. introduced a novel metabolic index, the CMI [12], which incorporates clinical indicators such as TG, HDL-C, and the WHtR. WHtR is primarily used to assess obesity, while the TG/HDL-C ratio provides a reflection of lipid levels, offering a more comprehensive evaluation of lipid metabolism. Several studies have demonstrated the clinical utility of the CMI in various metabolic disorders, including hypertension, ischemic stroke, obstructive sleep apnea, and hyperuricemia [13–16].

However, due to the limited number of studies, the relationship between albuminuria and CMI remains insufficiently explored and warrants further investigation. The aim of this study was to examine the association between CMI and albuminuria in participants of the NHANES.

## 2 Materials and methods

### 2.1 Survey description

The data for this study were provided by the NHANES, a population-based, cross-sectional study conducted by the National Center for Health Statistics (NCHS) to assess health and nutrition in the United States. NHANES employs a sophisticated, multistage, stratified probability sampling design conducted biennially, ensuring its representativeness of the U.S. population [17]. All research protocols for NHANES were approved by the NCHS Research Ethics Review Board, and informed consent was obtained from all participants. For individuals under the age of 16, consent was provided by their parents or legal guardians. Detailed NHANES study designs and data are publicly available at: https://www.cdc.gov/nchs/nhanes/.

### 2.2 Study population

**Fig 1:**
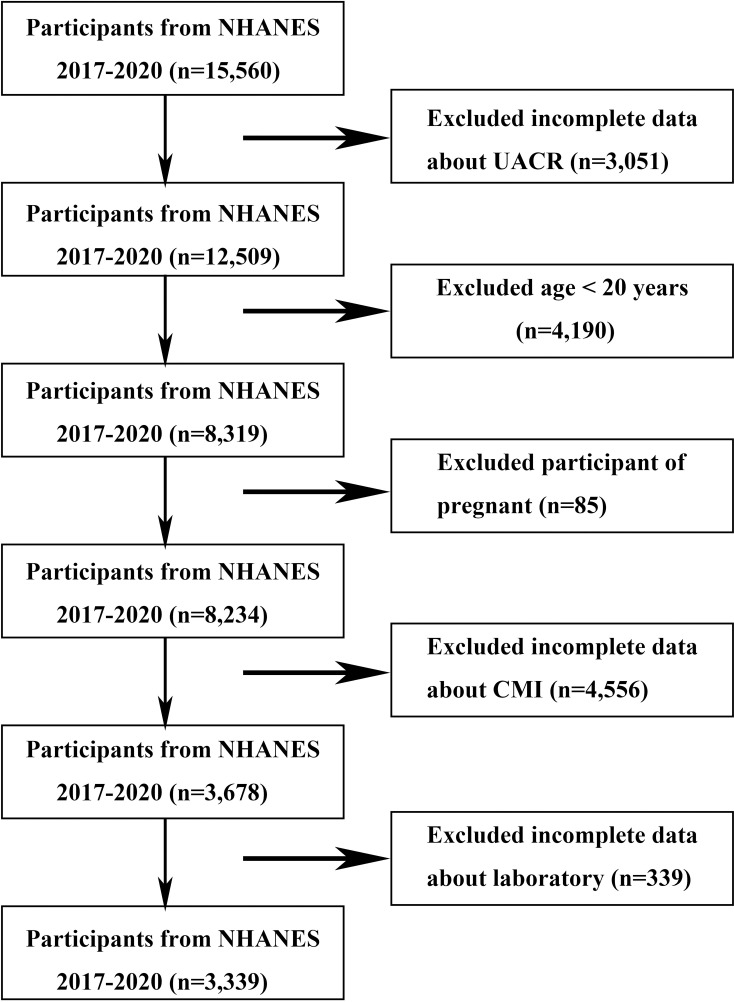
Flowchart of the sample selection from NHANES 2017–2020. Abbreviation: UACR, urinary albumin-creatinine ratio; CMI, cardiometabolic index.

This study utilized data from the 2017 to 2020 survey cycles of the NHANES to examine the association between CMI and albuminuria. Initially, 15,560 participants were enrolled. After excluding individuals with incomplete data on urinary albumin-to-creatinine ratio (UACR) (n =  3,051), participants aged < 20 years (n =  4,190), participants who were pregnant (n =  85), incomplete data on CMI (n =  4,556), and missing laboratory or clinical data (n =  339), a total of 3,339 participants remained in the final analysis (Fig 1).

### 2.3 Definition of CMI and albuminuria

CMI is a novel composite indicator that integrates lipid and obesity-related parameters, calculated from anthropometric data and laboratory measurements. Blood samples were typically collected either on the survey vehicle or at designated sampling points, where TG and HDL-C levels were measured. TG measurements were conducted only on samples from participants who had fasted for at least 9 hours prior to venipuncture, ensuring the preservation and stability of the samples. All samples were processed in accordance with standardized laboratory protocols to maintain data quality and comparability. Trained health technicians used mobile screening equipment to measure participants’ height and waist circumference. In our study, CMI was treated as the independent variable. The formula for calculating CMI is as follows: WHtR =  WC (cm)/ Height (cm); CMI =  TG (mmol/L)/HDL-C (mmol/L) ×  WHtR.

Albuminuria was defined as a UACR ≥  30 mg/g, based on previous research [18,19]. Participants provided urine samples at the end of the survey, which were then frozen at −20 °C and sent to a clinical laboratory. Urinary albumin levels were measured using solid-phase fluorescence immunoassay, while urinary creatinine levels were assessed using enzymatic methods. Quality assurance and control procedures for these specimens were strictly followed according to the NHANES Laboratory Procedures Manual.

### 2.4 Selection of covariates

The covariates included in the analysis were as follows: age (years), gender (male/female), body mass index (BMI, kg/m^2^), physical activity, hypertension, diabetes, smoking status (categorized as “no” for participants who had smoked fewer than 100 cigarettes in their lifetime and “yes” for those who had smoked more than 100 cigarettes), alcohol consumption (categorized as “no” for participants who consumed fewer than 12 alcoholic beverages in the past 12 months and “yes” for those who consumed 12 or more alcoholic beverages in the past 12 months), cardiovascular disease (CVD), history of stroke, systolic blood pressure (SBP, mmHg), diastolic blood pressure (DBP, mmHg), fasting plasma glucose (FPG, mg/dL), triglycerides (TG, mg/dL), total cholesterol (TC, mg/dL), high-density lipoprotein cholesterol (HDL-C, mg/dL), low-density lipoprotein cholesterol (LDL-C, mg/dL), serum creatinine (Scr, mg/dL), blood urea nitrogen (BUN, mg/dL), and serum uric acid (SUA, mg/dL). Age was categorized into two groups ( <60/ ≥ 60 years). BMI was classified into three categories ( <25/ ≥30 kg/m^2^), corresponding to normal weight, and overweight groups, respectively. Detailed measurements for all variables used in this analysis were obtained from NHANES database.

### 2.5 Statistical analysis

All statistical analyses were conducted following the guidelines recommended by the Centers for Disease Control and Prevention (CDC). Continuous variables were expressed as mean ±  standard deviation (SD) or median with interquartile ranges (IQR), while categorical variables were presented as frequencies and percentages. Differences in baseline characteristics across different groups of CMI quartiles were assessed using Student’s t-test for continuous variables and the chi-squared test for categorical variables. The association between CMI and albuminuria was evaluated using multivariate logistic regression in several models. Model 1: no adjustment for confounders. Model 2: adjusted for age, gender, alcohol consumption and smoking status. Model 3: adjusted for age, gender, alcohol consumption, smoking status, CVD, stroke, physical inactivity, SBP, DBP, ALT, AST, Scr, BUN and SUA. Further, the relationship between CMI and albuminuria was explored using smooth curve fitting. Given that certain covariates had missing values ranging from 0% to 15%, we excluded samples with missing data for those variables. Subgroup analyses were performed to examine the association between CMI and albuminuria across different subgroups, stratified by gender (male/female), age (<60/  ≥60 years), BMI (normal weight, overweight), diabetes status (yes/no), and hypertension status (yes/no). Interaction analysis was performed to evaluate the heterogeneity of associations between subgroups. All statistical analyses were performed using R version 4.1.2 and the EmpowerStats package (http://www.R-project.org). *P*-value < 0.05 was considered statistically significant.

## 3 Results

### 3.1 Baseline characteristics of participants

The study included 3,339 participants who met the inclusion criteria, with a mean age of 50.72 ±  17.17 years, and 49.81% of them were male. The prevalence of albuminuria was 12.79%. The clinical characteristics of the participants, stratified by albuminuria status, are presented in Table 1. The albuminuria group exhibited significantly higher values for age, diabetes, hypertension, CVD, stroke, physical inactivity, BMI, SBP, DBP, FPG, TG, BUN, Scr, SUA, and CMI (*P* <  0.05) compared to the normal albuminuria group. Additionally, the albuminuria group had significantly lower levels of TC, LDL-C, and HDL-C (*P* <  0.05).

**Table 1:** Baseline characteristics of included participants in the NHANES 2017–2020.

Variable	Overall	Albuminuria	Non-Albuminuria	P-value
	n = 3,339	n = 427	n = 2,912	
**Age (year)**	50.72 ± 17.17	59.04 ± 16.12	49.50 ± 16.98	<.001
**Gender (%)**				0.333
**Male**	1663 (49.81)	222 (51.99)	1441 (49.48)	
**Female**	1676 (50.19)	205 (48.01)	1471 (50.52)	
**Smoking status (%)**	1441 (43.16)	201 (47.07)	1240 (42.58)	0.080
**Alcohol consumption (%)**	2960 (88.65)	363 (85.01)	2597 (89.18)	0.011
**Diabetes (%)**	521 (15.60)	173 (40.52)	348 (11.95)	<.001
**Hypertension (%)**	1291 (38.66)	274 (64.17)	1017 (34.92)	<.001
**CVD (%)**	142 (4.25)	46 (10.77)	96 (3.30)	<.001
**Stroke (%)**	152 (4.55)	35 (8.20)	117 (4.02)	<.001
**Physical activity (%)**				0.024
**Vigorous**	869 (26.03)	93 (21.78)	776 (26.65)	
**Moderate**	738 (22.10)	87 (20.37)	651 (22.36)	
**Inactive**	1732 (51.87)	247 (57.85)	1485 (51.00)	
**BMI (kg/m2)**	29.82 ± 7.24	30.85 ± 7.92	29.67 ± 7.13	0.004
**SBP (mmHg)**	124.29 ± 19.42	136.95 ± 23.69	122.43 ± 17.98	<.001
**DBP (mmHg)**	75.07 ± 11.74	77.85 ± 13.74	74.66 ± 11.37	<.001
**FPG (mg/dl)**	113.08 ± 37.20	137.38 ± 64.94	109.52 ± 29.51	<.001
**TC (mg/dl)**	183.68 ± 40.81	177.82 ± 44.93	184.54 ± 40.10	0.001
**TG (mg/dl)**	105.21 ± 63.31	114.43 ± 65.22	103.85 ± 62.92	0.001
**LDL-C (mg/dl)**	109.04 ± 35.94	102.99 ± 38.08	109.93 ± 35.54	<.001
**HDL-C (mg/dl)**	53.60 ± 15.79	51.96 ± 17.24	53.83 ± 15.55	0.022
**ALT (IU/L)**	22.33 ± 19.63	22.29 ± 16.87	22.33 ± 20.00	0.966
**AST (IU/L)**	21.94 ± 15.01	23.13 ± 17.24	21.76 ± 14.65	0.119
**BUN (mg/dl)**	14.76 ± 5.65	17.91 ± 8.52	14.30 ± 4.93	<.001
**Scr (mg/dl)**	0.89 ± 0.43	1.10 ± 1.06	0.86 ± 0.21	<.001
**SUA (mg/dl)**	5.47 ± 1.45	5.79 ± 1.74	5.43 ± 1.39	<.001
**CMI**	1.43 ± 1.24	1.67 ± 1.32	1.39 ± 1.22	<.001

Abbreviations: CVD, cardiovascular disease; BMI, body mass index; SBP, systolic blood pressure; DBP, diastolic blood pressure; FPG, fasting plasma glucose; TC, total cholesterol; TG, triglyceride; LDL-C, low-density lipoprotein cholesterol; HDL-C, high-density lipoprotein cholesterol; ALT, aspartate aminotransferase; AST, aspartate aminotransferase; BUN, blood urea nitrogen; Scr, serum creatinine; SUA, serum uric acid; CMI, cardiometabolic index.

Participants were categorized into four groups based on the quartiles of CMI: Quartile 1 (CMI ≤  0.577), Quartile 2 (0.577 < CMI ≤  1.04), Quartile 3 (1.04 < CMI ≤  1.84), and Quartile 4 (CMI >  1.84). Compared to the Quartile 1 (lowest CMI) group, individuals in Quartiles 2, 3, and 4 were significantly more likely to be male, age, smokers, and have higher prevalence of diabetes, hypertension, CVD, stroke, physical inactivity, BMI, FPG, TC, TG, LDL-C, ALT, BUN, Scr, and SUA, while they had lower levels of HDL-C and engaged in less vigorous physical activity (P <  0.05). Notably, the risk of albuminuria increased progressively with higher CMI levels: 7.78% in Quartile 1, 13.43% in Quartile 2, 12.93% in Quartile 3, and 17.01% in Quartile 4 (*P* <  0.001). The detailed clinical characteristics of the participants are provided in Table 2.

**Table 2:** Baseline characteristics of the study population according to CMI quartiles.

Variable	Overall	CMI in Quartile				P-value
		Quartile 1	Quartile 2	Quartile 3	Quartile 4	
	n = 3,339	n = 835	n = 834	n = 835	n = 835	
Age (year)	50.72 ± 17.17	46.25 ± 18.32	50.99 ± 17.03	52.82 ± 16.59	52.83 ± 15.81	<.001
Gender (%)						<.001
Male	1663 (49.81)	357 (42.75)	398 (47.72)	418 (50.06)	490 (58.68)	
Female	1676 (50.19)	478 (57.25)	436 (52.28)	417 (49.94)	345 (41.32)	
Smoking status (%)	1441 (43.16)	323 (38.68)	340 (40.77)	359 (42.99)	419 (50.18)	<.001
Alcohol consumption (%)	2960 (88.65)	753 (90.18)	726 (87.05)	744 (89.10)	737 (88.26)	0.226
Diabetes (%)	521 (15.60)	41 (4.91)	103 (12.35)	155 (18.56)	222 (26.59)	<.001
Hypertension (%)	1291 (38.66)	221 (26.47)	307 (36.81)	362 (43.35)	401 (48.02)	<.001
CVD (%)	142 (4.25)	13 (1.56)	34 (4.08)	39 (4.67)	56 (6.71)	<.001
Stroke (%)	152 (4.55)	27 (3.23)	39 (4.68)	40 (4.79)	46 (5.51)	0.155
Physical activity (%)						0.006
Vigorous	869 (26.03)	242 (28.98)	222 (26.62)	207 (24.79)	198 (23.71)	
Moderate	738 (22.10)	191 (22.87)	184 (22.06)	156 (18.68)	207 (24.79)	
Inactive	1732 (51.87)	402 (48.14)	428 (51.32)	472 (56.53)	430 (51.50)	
BMI (kg/m2)	29.82 ± 7.24	25.28 ± 5.27	29.02 ± 6.47	31.45 ± 6.82	33.53 ± 7.49	<.001
SBP (mmHg)	124.29 ± 19.42	120.87 ± 19.55	124.47 ± 19.62	125.96 ± 20.04	125.86 ± 17.98	<.001
DBP (mmHg)	75.07 ± 11.74	72.34 ± 11.77	75.08 ± 11.90	75.93 ± 11.57	76.91 ± 11.25	<.001
FPG (mg/dl)	113.08 ± 37.20	100.26 ± 14.68	107.68 ± 28.72	114.79 ± 33.67	129.58 ± 53.83	<.001
TC (mg/dl)	183.68 ± 40.81	177.99 ± 39.33	180.50 ± 38.12	185.12 ± 41.39	191.09 ± 43.07	<.001
TG (mg/dl)	105.21 ± 63.31	49.65 ± 15.10	76.49 ± 19.53	107.56 ± 25.08	187.09 ± 64.60	<.001
LDL-C (mg/dl)	109.04 ± 35.94	99.03 ± 31.75	108.97 ± 33.38	115.23 ± 37.52	112.94 ± 38.57	<.001
HDL-C (mg/dl)	53.60 ± 15.79	69.02 ± 17.37	56.23 ± 11.14	48.39 ± 8.81	40.74 ± 7.51	<.001
ALT (IU/L)	22.33 ± 19.63	18.02 ± 12.84	20.17 ± 14.01	23.68 ± 27.79	27.42 ± 18.93	<.001
AST (IU/L)	21.94 ± 15.01	21.79 ± 14.82	21.08 ± 12.11	22.06 ± 20.07	22.82 ± 11.46	0.123
BUN (mg/dl)	14.76 ± 5.65	14.02 ± 4.87	14.45 ± 5.16	14.63 ± 5.25	15.96 ± 6.90	<.001
Scr (mg/dl)	0.89 ± 0.43	0.86 ± 0.41	0.89 ± 0.33	0.87 ± 0.24	0.93 ± 0.65	0.006
SUA (mg/dl)	5.47 ± 1.45	4.83 ± 1.27	5.34 ± 1.32	5.71 ± 1.40	6.02 ± 1.51	<.001
Albuminuria (%)	427 (12.79)	65 (7.78)	112 (13.43)	108 (12.93)	142 (17.01)	<.001

**Abbreviations:** CVD, cardiovascular disease; BMI, body mass index; SBP, systolic blood pressure; DBP, diastolic blood pressure; FPG, fasting plasma glucose; TC, total cholesterol; TG, triglyceride; LDL-C, low-density lipoprotein cholesterol; HDL-C, high-density lipoprotein cholesterol; ALT, aspartate aminotransferase; AST, aspartate aminotransferase; BUN, blood urea nitrogen; Scr, serum creatinine; SUA, serum uric acid.

### 3.2 Association between CMI and albuminuria

The association between CMI and the risk of albuminuria is summarized in Table 3. In the crude model 1, the odds ratio (OR) was 1.17 (95% CI: 1.09–1.25), demonstrating a significant positive relationship between CMI and albuminuria. In adjusted model 2, which accounted for age, gender, alcohol consumption and smoking status, this positive association remained significant (OR =  1.16, 95% CI: 1.07–1.25). Further adjustments in adjusted model 3, incorporating CVD, stroke, physical inactivity, SBP, DBP, ALT, AST, Scr, BUN and SUA, continued to show a robust positive correlation between CMI and albuminuria (OR =  1.16, 95% CI: 1.07–1.27). Sensitivity analyses using CMI as a categorical variable (quartiles) revealed that individuals in the highest CMI quartile had a 126% higher risk of albuminuria compared to those in the lowest quartile (OR =  2.26, 95% CI: 1.58–3.23; *P* for trend <  0.05).

**Table 3:** The association between CMI and albuminuria.

CMI	OR (95%CI), P value		
	Crude model 1	Adjusted model 2	Adjusted model 3
Continuous	1.17 (1.09 ~ 1.25)	1.16 (1.07 ~ 1.25)	1.16 (1.07 ~ 1.27)
Categories			
Quartile 1	1.00 (Reference)	1.00 (Reference)	1.00 (Reference)
Quartile 2	1.84 (1.33 ~ 2.54)	1.62 (1.17 ~ 2.26)	1.72 (1.21 ~ 2.43)
Quartile 3	1.76 (1.27 ~ 2.43)	1.48 (1.07 ~ 2.06)	1.64 (1.14 ~ 2.34)
Quartile 4	2.43 (1.78 ~ 3.31)	2.06 (1.50 ~ 2.84)	2.26 (1.58 ~ 3.23)
P for trend	<0.001	<0.001	<0.001

95% CI: 95% confidence interval. OR: Odds ratio.

Model 1: No covariates were adjusted. Adjusted model 2: Adjusted for gender, age, alcohol consumption and smoking status. Adjusted model 3: Adjusted for gender, age, alcohol consumption, smoking status, CVD, stroke, physical inactivity, SBP, DBP, ALT, AST, Scr, BUN and SUA.

### 3.3 Subgroup analysis

**Fig 2:**
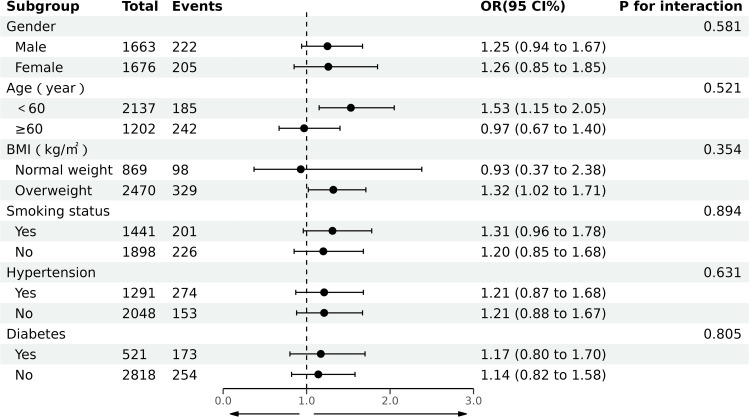
Association between CMI and albuminuria in subgroup and interactive analyses. In the multivariable logistic regression models, covariates were adjusted as adjusted model 3 in previous analyses except for subgroup variables.

Fig 2 illustrates the subgroup analyses examining the association between CMI and albuminuria. To assess whether this relationship remains consistent across various population subgroups, stratified analyses were conducted based on age, gender, BMI, smoking status, hypertension, and diabetes, accompanied by interaction tests. The results demonstrated a significant association between CMI and albuminuria in subgroups characterized by age < 60 years and overweight status (all *P* <  0.05). In contrast, within the subgroups defined by gender, smoking status, hypertension, and diabetes, CMI showed a positive but statistically non-significant association with albuminuria (all *P* >  0.05). Furthermore, interaction tests indicated no significant modifying effects of age, gender, BMI, smoking status, hypertension, or diabetes on the relationship between CMI and albuminuria (all *P* for interaction >  0.05).

### 3.4 Smoothed curve fitting and threshold effect analysis

**Fig 3:**
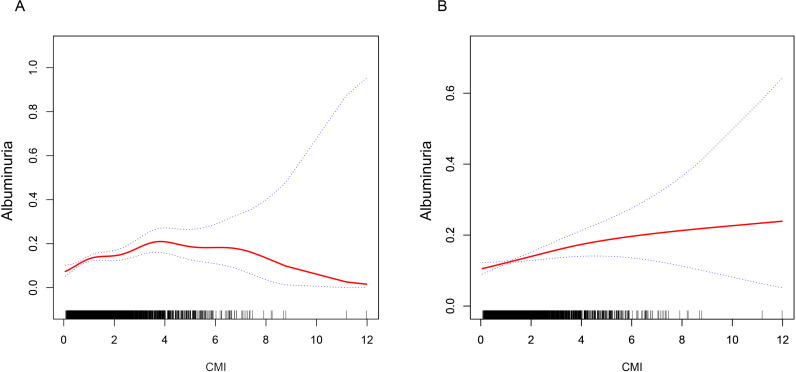
The relationship between CMI and albuminuria was analyzed by smoothed curve fitting. A, Smoothed curve fitting analysis of the relationship between CMI and albuminuria without correction for any factor. B, after adjusting for gender, age, alcohol consumption, smoking status, CVD, stroke, physical inactivity, SBP, DBP, ALT, AST, Scr, BUN and SUA, the relationship between CMI and albuminuria was analyzed by smooth curve fitting.

Using smoothed curve fitting, we examined the connection between CMI and albuminuria in more detail. According to the findings, there was a positive correlation between CMI and the probability of developing albuminuria (OR =  1.17, 95% CI: 1.09–1.25) (Fig 3A). Then after controlling for the covariates of age, gender, alcohol consumption, smoking status, CVD, stroke, physical inactivity, SBP, DBP, ALT, AST, Scr, BUN and SUA, we found J-shaped associations between CMI and the probability of albuminuria (Fig 3B), and subsequently analyzed the threshold effect using a two-stage linear regression model, with a calculated the inflection point was 0.91. On the left side of the inflection point, there was a significant correlation between CMI and albuminuria (OR =  2.24, 95% CI: 1.22–4.12), suggesting that CMI was an independent risk factor for albuminuria when CMI was lower than 0.92, and suggesting that the CMI metrics could be used for early prediction and assessment of albuminuria (Table 4).

**Table 4.** Threshold effect analysis of CMI on albuminuria using a linear regression model.

CMI	Adjusted OR (95%CI), *P* value
Standard linear model	1.17 (1.07, 1.27) 0.0004
Two-piecewise linear model	
Inflection point	0.92
OR1 (CMI < 0.92)	2.24 (1.22, 4.12) 0.0094
OR2 (CMI > 0.92)	1.11 (1.01, 1.22) 0.0392
Log likelihood ratio	0.031

95% CI: 95% confidence interval. OR: Odds ratio. Adjusted for gender, age, alcohol consumption, smoking status, CVD, stroke, physical inactivity, SBP, DBP, ALT, AST, Scr, BUN and SUA.

## 4 Discussion

To the greatest of our knowledge, this is the first study in the United States to assess the relationship between CMI and albuminuria in a population of all ages. The authors of a cross-sectional research with 3, 339 participants discovered a positive correlation between CMI and albuminuria, indicating a steady rise in albuminuria likelihood with rising CMI levels. The similarity of this connection was demonstrated by subgroup analysis and interaction tests in various demographic contexts.

Excessive fat accumulation and abdominal obesity have been shown to induce renal inflammation and oxidative stress, promote proteinuria, and accelerate the progression of kidney disease [20]. Lifestyle interventions are recognized as essential strategies for alleviating the burden of CKD [21]. Recent studies have highlighted the benefits of adopting healthy lifestyle practices, including dietary modifications, weight management, and regular physical activity, in reducing mortality and cardiovascular risk among CKD patients [22–25]. However, the implementation of lifestyle interventions remains challenging due to factors such as behavioral heterogeneity, variability in reference populations, and the complexity of CKD progression classification [26]. Consequently, the development of effective predictive markers for individuals in the preclinical stage is of critical importance. In this study, we identified a significant association between the CMI and proteinuria. As CMI integrates information on body fat distribution and lipid metabolism abnormalities, it provides a more comprehensive assessment of metabolic dysfunction [27,28]. These findings underscore the strong relationship between CMI and the risk of albuminuria, highlighting its potential utility in identifying high-risk populations for CKD and contributing to early screening and prevention efforts.

Obesity may be a risk factor for both CKD and ESRD, according to epidemiologic studies [29,30]. Of them, subcutaneous adipose tissue is regarded as benign or protective, but visceral fat accumulation is the primary pathogenic state of obesity [31,32]. BMI and WC are easy to examine and have been widely used to define obesity and abdominal obesity [33]. The Look AHEAD study demonstrated that compared with the lowest quartile, BMI (OR =  1.72, 95% CI: 1.40–2.11) and WC (OR =  1.75, 95% CI: 1.42–2.15) in the highest quartile were significantly associated with albuminuria [34]. Postorino et al. discovered that WC was a direct predictor of both cardiovascular and all-cause mortality in ESRD patients, indicating that abdominal obesity is the root cause of a high risk of a bad prognosis [35]. Nevertheless, it is not possible to distinguish between visceral and subcutaneous fat mass using BMI or WC [36]. Because subcutaneous and visceral adipose tissue differ greatly in functional significance, anthropometric data alone are insufficient for accurate risk assessment of obesity [37]. As a result, quicker obesity diagnosis will be possible using image-based body fat evaluation. Computed Tomography (CT) and Magnetic resonance imaging (MRI) can accurately measure visceral fat area, generate high-resolution images, and have high reproducibility [38,39]. However, CT is expensive and exposes patients to radiation, and there is a lack of research on related techniques to assess visceral fat. It has been stated that CMI, a recently created tool for evaluating a patient’s fat distribution (a model that incorporates anthropometrics and blood metabolic data), is being investigated in a number of domains, particularly in connection to disorders linked to metabolism. In the current study, we found a strong association between CMI and albuminuria. However, the underlying mechanisms linking CMI and albuminuria remain unclear. Previous studies have suggested that CMI, as a more appropriate marker for assessing obesity, may explain the association. First, when the production of lipids is higher than the capacity of fat storage, it results in the deposition of lipids in tissues and organs such as kidneys [40]. Lipid accumulation can induce inflammation, oxidative stress, and autophagy through multiple signaling pathways, leading to massive proliferation of glomerular basement membrane cells, exacerbating glomerulosclerosis and tubulointerstitial injury, and facilitating the production of proteinuria, leading to progressive kidney injury [41–43]. Secondly, fat distribution is also important for renal function impairment. It has been shown that subjects with a centralized fat distribution, whether lean, overweight, or obese, have a greater risk of reduced glomerular filtration rate [44]. Central body fat distribution is associated with altered renal hemodynamic characteristics, which is caused by an imbalance in the vasodilatory balance between afferent and efferent arterioles, leading to an increase in the filtration fraction. This may play a role in susceptibility to and progression of chronic kidney injury [45]. Thirdly, urinary protein excretion rate is used as a marker for early screening of patients at high risk of kidney disease, but available evidence suggests that UACR is less sensitive in predicting 24-h total proteinuria, is no more effective in predicting patient-related outcomes, fails to detect a large number of patients with a small amount of proteinuria, and is more costly [46–49]. CMI, as an indicator that takes into account both lipid and fat distribution, may predict the progression of renal disease through the mechanisms described above, and also complements proteinuria excretion rate as a predictor of early renal disease.

Our study has several strengths. By utilizing data from NHANES, a nationally representative population-based sample collected through standardized procedures, the study sample is highly representative. Additionally, the authors accounted for multiple confounding factors, enhancing the reliability and robustness of the findings. However, the limitations of this study cannot be overlooked. First, despite adjusting for various potential confounders, albuminuria is influenced by numerous factors, and the impact of other unmeasured confounders cannot be entirely ruled out. Second, due to the cross-sectional design of this study, we were unable to establish a definitive causal relationship between CMI and albuminuria. Therefore, further longitudinal studies are needed to confirm whether CMI can serve as a reliable screening tool for the prevention of early-stage renal disease.

## 5 Conclusion

This study indicates that elevated CMI levels are significantly associated with an increased risk of albuminuria and may serve as a promising screening marker for the early detection and prevention of kidney disease.
